# What are the perceptions about running and knee joint health among the public and healthcare practitioners in Canada?

**DOI:** 10.1371/journal.pone.0204872

**Published:** 2018-10-01

**Authors:** Jean-Francois Esculier, Natasha M. Krowchuk, Linda C. Li, Jack E. Taunton, Michael A. Hunt

**Affiliations:** 1 Department of Physical Therapy, Faculty of Medicine, University of British Columbia, Vancouver, British Columbia, Canada; 2 Arthritis Research Canada, Richmond, British Columbia, Canada; 3 Department of Family Medicine, Faculty of Medicine, University of British Columbia, Vancouver, British Columbia, Canada; EHESP Paris, FRANCE

## Abstract

**Objectives:**

To evaluate the perceptions of the general public and healthcare practitioners (HCP) in Canada about the relationship between running and knee joint health, and to explore HCP`s usual recommendations to runners with knee osteoarthritis (KOA).

**Methods:**

Non-runners and runners (with and without KOA) and HCP completed an online survey regarding the safety of running for knee joint health. HCP also provided information related to usual clinical recommendations. Proportions of agreement were compared between non-runners and runners.

**Results:**

A total of 114 non-runners, 388 runners and 329 HCP completed the survey. Overall, running was perceived as detrimental for the knee joint by 13.1% of the general public, while 25.9% were uncertain. More uncertainty was reported regarding frequent (33.9%) and long-distance (43.6%) running. Statistical analyses revealed greater proportions of non-runners perceiving running negatively compared with runners. Overall, 48.4% believed that running in the presence of KOA would lead to disease progression, while 53.1% believed running would lead to premature arthroplasty. In HCP, 8.2%, 9.1% and 22.2% perceived that running in general, running frequently, or running long-distances are risk factors for KOA, respectively. 37.1% and 2.7% of HCP typically recommended patients with KOA to modify their running training or to quit running, respectively.

**Conclusion:**

High rates of uncertainty among the general public and HCP in Canada outline the need for further studies about running and knee joint health. Filling knowledge gaps will help inform knowledge translation strategies to better orientate the general public and HCP about the safety of running for knee joint health.

## Introduction

Knee osteoarthritis (KOA) is a leading cause of long-term disability in Canada and across the world, resulting in chronic pain and activity limitations, and eventually decreased quality of life [1]. This condition also bears significant economic burden due to treatments, loss of productivity and indirect healthcare costs [1]. KOA is thought to develop because of a variety of risk factors including advancing age, obesity, previous trauma and genetics [2]. Regular physical activity can help in reducing the incidence of KOA and its economic burden, in part because of its beneficial effects on weight control [3, 4]. In addition, physical activity and exercise have been widely recognized as essential components of clinical management of people with KOA [5, 6]. Nevertheless, adherence to recommendations in the population largely depends on perceptions about the benefits of specific types of physical activity as well as on barriers to participation.

Only 13% of individuals with KOA meet weekly physical activity guidelines of at least 150 minutes of moderate to vigorous activity and only 19% reach 10,000 daily steps [7]. It has been suggested that patients with KOA perceive lower effectiveness of physical activity and exercises for their condition in comparison with healthcare practitioners (HCP) [8]. Hence, to optimize adherence to physical activity recommendations, it is important to tailor advice to patients so that meaningful and motivating activities are prescribed [9].

Since recreational running is a very popular form of activity throughout the lifespan, it potentially represents an interesting option for many to meet the physical activity requirements for effective prevention or clinical management of KOA. Running is known to provide a number of physical and psychological benefits regardless of age or health condition [10, 11], and is highly accessible given that it requires little to no equipment. However, running has traditionally been associated with a negative outcome for knee joint health [12], since the knee represents one of the most frequently injured body part in runners [13, 14]. Nonetheless, recent evidence is inconclusive about running being a definite causative factor of KOA, as specific training parameters may influence prevalence more than others [15]. For example, Alentorn-Geli et al. recently suggested that recreational running was linked with lower rates of KOA, while competitive running was associated with greater rates [15]. A dearth in knowledge about the effects of running on KOA, both in terms of prevention and treatment, currently limits the ability to provide clear clinical recommendations. However, research on the topic is growing rapidly, and knowledge translation strategies to the public and HCP will likely be indicated in the near future.

To date, it remains unknown how the general population and HCP perceive running with respect to knee joint health. Documenting beliefs in the general population regarding running as a risk factor for developing KOA is needed to assist in promoting meaningful activities for the prevention of KOA. It is also necessary to understand the perceptions about the appropriateness of running in individuals with pre-existing KOA. Indeed, insights on beliefs may help in understanding potential demographic, physical, psychosocial or environmental barriers to running and physical activity in general in those with KOA [16] and inform future clinical recommendations. Furthermore, since recommendations from HCP likely influence choices of recreational activities in the general population, an assessment of HCP`s beliefs and recommendations regarding running and knee joint health is warranted. Conflicting advice from HCP regarding physical activity has previously been identified as a factor that causes uncertainty and confusion in individuals with KOA [17], which could potentially restrict participation. A clearer understanding of HCP’s perceptions could assist in designing future knowledge translation strategies to clinicians, as well as in planning further studies about running and knee joint health.

Therefore, the objectives of this survey study were to evaluate the perceptions of the public and HCP about the relationship between running and knee joint health and explore HCP`s recommendations to runners with KOA. We also sought to evaluate if perceptions and recommendations from HCP differ based on their own running habits.

## Materials and methods

### Participants

A web-based, descriptive, cross-sectional survey was conducted among the Canadian population between September 11, 2017 and January 30, 2018. A general recruitment campaign was made through social media advertisements (Facebook, Twitter), email blasts to members of HCP provincial and national associations (physicians, physiotherapists, chiropractors, athletic therapists), associations of arthritis research consumers, sports medicine clinics, as well as to running groups. Once they provided consent to participate, respondents self-identified into one of five subgroups based on their profile: (1) non-runners without KOA (NRUN); (2) non-runners who have received a diagnosis of KOA (NRUN-OA); (3) runners without KOA (RUN); (4) runners who have received a diagnosis of KOA (RUN-OA); and (5) HCP of various professional backgrounds. Members of the general public (subgroups 1–4) had to be aged 40 years and older, while HCP (subgroup 5) could be of any age. Those who self-identified as HCP did not need to be currently licensed or practicing but did need to have previous formal training in their discipline. All participants were living in Canada, had to understand English or French, and had to have access to the internet to fill out the survey. This study was approved by the institutional ethics review board and informed consent was obtained online prior to survey commencement.

### Study design

Participation in this study required the completion of a single round of questions in English or French, the two official languages in Canada. The online survey was designed and conducted using a structured sequence of four steps. The following process ensured that adequate information was collected, that questions could be easily read and understood by individuals with and without medical background and knowledge, and that the English and French versions were assessing the same constructs so that responses could be combined in overall analyses.

#### Step 1. Designing the first version of the questionnaire and assessment of face validity

The research team designed the initial set of core questions in English to capture information on demographics, current health status and level of physical activity, as well as perceptions about running and knee joint health. Additional questions were then added to specifically target the different subgroups and were only provided to individuals in each subgroup as appropriate. Once completed, the first version of the questionnaire was sent to individuals external to the research team for peer-review and assessment of face validity. Overall, three HCP who were also runners (one sports medicine physician, two physiotherapists) and three patient partners with KOA gave feedback on readability and appropriateness, and provided suggestions on the pertinence and wording of the individual survey questions. These six reports allowed us to validate items related to running, KOA and HCP recommendations.

#### Step 2. Designing the final version

The first version was modified considering comments from external reviewers and further input from the research team. The final version of the questionnaire included 20 items for NRUN and NRUN-OA, 26 items for RUN, 28 items for RUN-OA and 26 items for HCP (see S1 File). Five sections (18 questions) were identical for all subgroups, with additional subgroup-specific questions as described below. The first section gathered information on demographics (age, sex, education, height and mass). In the second section, all individuals were asked about their general health status and previous history of traumatic knee injuries. The third section included questions pertaining to the level of physical activity and participation in different types of recreational physical activity. Section 4 included questions about the perception of running in general, and if running often or long distances were perceived as risk factors for developing KOA. The fifth section assessed the perception about the appropriateness of running by individuals with diagnosed KOA, and if doing so would accelerate the need for joint replacement. All subgroups of non-runners and runners also provided details on any advice that they had received regarding running and knee joint health, and their sources of information.

Subgroup-specific questions included surveying those in the NRUN group if one of the reasons that they were not running was to avoid developing KOA. We also questioned NRUN-OA about their previous running habits (if any), and if reasons for stopping included their diagnosis of KOA. Details about running history (years of running, longest distance) and habits over the previous six months (frequency, distance, speed) were asked to the runner subgroups (RUN, RUN-OA). Additional items documented the perception of current runners about whether a future diagnosis of KOA would make them change their running habits (RUN) or if their previous diagnosis of KOA prompted them to modify their running habits (RUN-OA). Finally, we asked HCP to provide details about their professional background. They also provided insight on their personal clinical recommendations to runners with KOA (modification or cessation of running training), and whether they would recommend running to individuals who previously underwent total knee arthroplasty.

#### Step 3. Translation of the questionnaire and approval of the French version

The final version of the English questionnaire was translated into French by a bilingual physiotherapist (JFE). Then, the translated version was verified by an external bilingual physiotherapist with a special interest in running and KOA. Specifically, the reviewer ensured that the meaning of questions and answers was similar between both languages. He also verified orthography and grammar of the questionnaire. Since all questions and available responses of the French translation were deemed appropriate by the external reviewer, no further changes were made to the questionnaire after review.

#### Step 4. Administration of the questionnaire

Online versions of the English and French versions of the questionnaire were built using the FluidSurvey survey system (www.fluidsurveys.com). After clicking on the hyperlink leading to the survey webpage, potential participants selected their preferred language. Thereafter, a short plain language statement outlining the purpose of the study and the inclusion criteria was presented. Consent to participate was obtained by clicking a button which directed respondents to the full survey. Selection of the appropriate subgroup yielded the corresponding appropriate set of questions, as described above.

### Data analysis

All survey data were exported from the FluidSurvey website and compiled into a Microsoft Excel spreadsheet (Microsoft Corporation, Redmond, WA, USA). Descriptive statistics were computed for continuous (mean) and categorical (frequency) variables of interest and subgroup demographics. Proportions of agreement with survey items were supplemented with confidence intervals (95% C.I.). The four general public subgroups (NRUN, NRUN-OA, RUN, RUN-OA) were compared to each other, while HCP were further categorized into subgroups of professions for descriptive purposes. Given that clinical recommendations from HCP could potentially be affected by personal biases, we also compared HCP recommendations based on current running status. One-way analyses of variance (ANOVA) were used to compare between-group demographics characterized by continuous variables (self-reported age and body mass index). Proportions were compared between subgroups for all categorical variables using Chi-squared tests for contingency tables. The alpha level was set at 0.05. When between-subgroup proportions were significantly different, Bonferroni-adjusted post-hoc comparisons were conducted. All statistical analyses were performed using the Statistical Package for Social Sciences version 22 (SPSS; IBM, Armonk, NY).

## Results

### Respondents’ characteristics

A total of 114 non-runners (NRUN, NRUN-OA; 71.9% female, mean age = 61.4±11.0 years, mean BMI = 26.6±5.2 kg/m^2^), 388 runners (RUN, RUN-OA; 58.0% female, mean age = 51.0±7.9 years, mean BMI = 23.8±3.2 kg/m^2^) and 329 HCP (61.1% female, mean age = 38.6±11.2 years, mean BMI = 24.2±4.1 kg/m^2^) completed the survey. Subgroup socio-demographic characteristics for runners and non-runners are presented in Table 1. RUN and RUN-OA showed significantly lower BMI and significantly better self-perceived health status than NRUN and NRUN-OA, but were also significantly younger (all *P*<0.001; Table 1). Table 2 presents socio-demographics and professional characteristics of HCP.

**Table 1:** Demographics of non-runners and runners responding to the survey (N = 502).

	NRUN N = 52	NRUN-OA N = 62	RUN N = 338	RUN-OA N = 50	*P*-value
Sex (%)					0.037
Females	75.0	69.4	57.1	64.0	
Males	25.0	30.6	42.9	36.0	
Age (years)	58.6 (11.4)	63.7 (10.2)	50.4 (7.9)	54.8 (7.2)	**<0.001**
BMI (kg/m^2^)	25.6 (4.9)	27.5 (5.4)	23.8 (3.2)	23.7 (3.1)	**<0.001**
Level of education (%)					0.009
Less than high school completion	0	0	0.6	0	
High school	7.7	12.9	3.0	14.0	
Trades certificate, vocational school diploma, apprenticeship	17.3	9.7	7.1	2.0	
Non-university certificate below Bachelor's level	17.3	19.4	27.2	18.0	
Bachelor's degree	23.1	33.9	34.6	34.0	
Masters degree	23.1	17.7	20.7	28.0	
Doctorate degree	11.5	6.5	6.8	4.0	
Self-reported health status (%)					**<0.001**
Excellent	9.6	11.3	45.0	34.0	
Very good	50.0	38.7	44.7	54.0	
Good	26.9	32.3	9.8	10.0	
Fair	11.5	11.3	0.6	2.0	
Poor	1.9	6.5	0	0	
History of knee trauma (%)	75.0	45.2	78.4	40.0	**<0.001**

**Table 2:** Demographics of HCP responding to the survey (n = 329).

	Medical Doctors N = 27	Physiotherapists N = 148	Chiropractors N = 14	Athletic Therapists N = 85	Other N = 55
Sex (%)					
Females	44.4	62.8	50.0	61.2	67.3
Males	55.6	37.2	50.0	38.8	32.7
Age (years)	46.5 ± 12.6	39.0 ± 10.2	37.6 ± 8.6	36.2 ± 10.4	37.7 ± 13.4
Primary area of practice (%)					
Orthopaedics	7.4	68.9	0	17.6	21.8
Rheumatology	7.4	4.1	0	0	1.8
General practice	14.8	4.1	14.3	22.4	14.5
Sports medicine	55.6	10.8	57.1	45.9	16.4
Other	14.8	12.2	28.6	14.1	45.5
Years of professional practice (%)					
< 1 year	0	3.4	7.1	10.6	16.4
At least 1 year, but less than 3 years	14.8	8.8	7.1	11.8	16.4
At least 3 years, but less than 5 years	3.7	8.1	7.1	12.9	7.3
At least 5 years, but less than 10 years	18.5	15.5	21.4	22.4	18.2
At least 10 years, but less than 20 years	22.2	36.5	42.9	24.7	21.8
> 20 years	40.7	27.7	14.3	17.6	20.0
Diagnosed with KOA (%)	18.5	5.4	7.1	7.1	5.5
History of running (%)					
Currently runs regularly (>1 / week)	48.1	66.2	78.6	23.5	61.8
Currently runs infrequently (<1 / week)	11.1	5.4	7.1	14.1	7.3
Not currently running but ran in the past	29.6	18.9	14.3	43.5	25.5
Never ran regularly or infrequently	11.1	9.5	0	18.8	5.5

### Decisions about running relative to KOA

Detailed point-estimate data for proportions of agreement with survey items and 95% C.I. are provided in Supporting information (S2–S3 Files). Across all respondents (general public and HCP), 592 (71.2%) were currently runners and 234 (28.8%) were non-runners. Among the 29 respondents from NRUN-OA reporting a previous history of running, 5 (17.2%) quit running specifically due to their diagnosis of KOA. In the NRUN subgroup, 9 (17.3%) stated that they were not running due to fear of developing KOA, and 9 others (17.3%) were uncertain. Of those currently running but without a diagnosis of KOA, only 16 (4.7%) indicated they would quit running if diagnosed with KOA, while 56.8% would reduce running distances, 34.9% would reduce speed, and 26.9% would run less often. Overall, 80.2% declared that they would modify their running habits if diagnosed with KOA. Among runners with KOA, 6 (12.0%) indicated that they temporarily stopped running immediately upon their diagnosis of KOA. While 21 runners (42.0%) did not change their running habits at all, 26.0% reduced running distances, 22.0% reduced frequency and 14.0% reduced speed. A total of 332 (66.1%) members of the general public have received information specifically regarding running and joint health; the most frequent sources were from a physiotherapist (32.9%), the Internet (20.1%), friends and family (15.3%), a family doctor (13.9%) and scientific literature (13.1%).

### Perception about running and development of KOA

**Fig 1:**
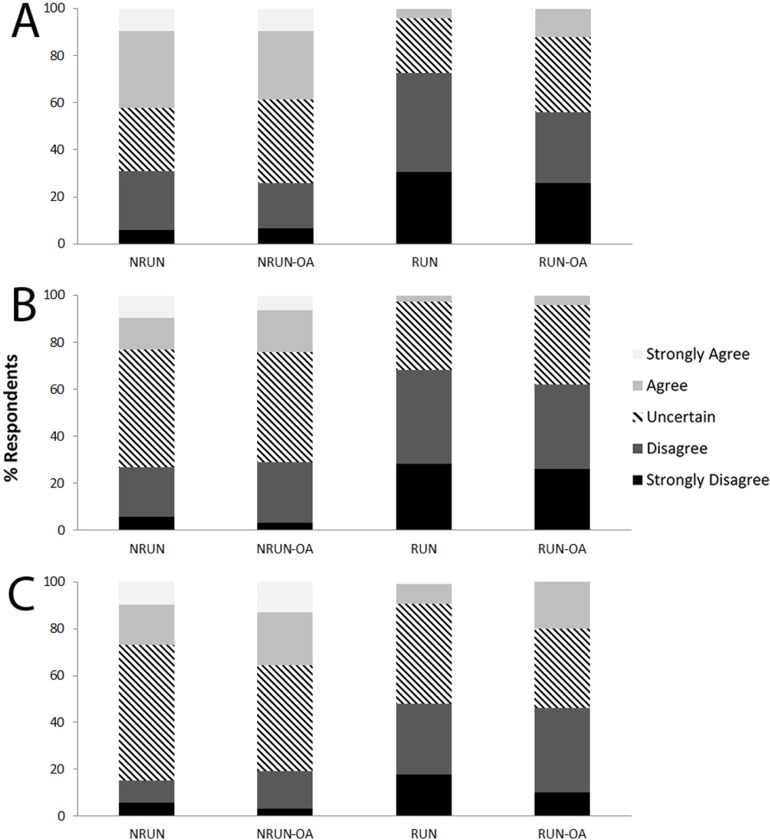
Perception of non-runners and runners about running and the development of knee osteoarthritis. (A) “In general, I see regular running as an activity that hurts the knee joint”; (B) “Frequent running can lead to getting knee osteoarthritis”; (C) “Running long distances (such as marathons and ultra-marathons) can lead to getting knee osteoarthritis”.

**Fig 2:**
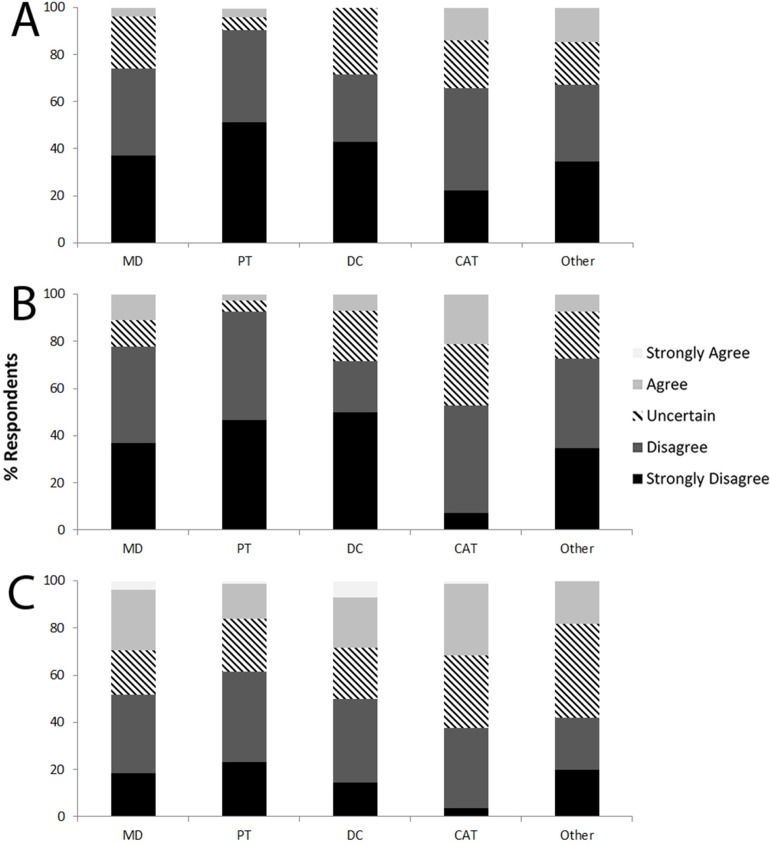
Perception of HCP about running and the development of knee osteoarthritis. (A) “In general, I see regular running as an activity that hurts the knee joint”; (B) “Frequent running can lead to getting knee osteoarthritis”; (C) “Running long distances (such as marathons and ultra-marathons) can lead to getting knee osteoarthritis”.

Overall, regular running was perceived as an activity that hurts the knee joint by 13.1% of respondents from the public, while 25.9% were uncertain (Fig 1A). Analyses revealed that a significantly greater proportion (*P*<0.001) of respondents from NRUN (42.3%) and NRUN-OA (38.7%) tended to have a negative perception compared with RUN (4.1%). A total of 8.2% of HCP agreed that regular running was detrimental for knee joint health, while 78.1% disagreed (Fig 2A). A negative perception was reported by 3.9% of HCP who run and by 15.2% of HCP who didn’t run (*P*<0.001).

Running frequently was seen by only 7.6% of members of the public as an activity that leads to KOA, but 33.9% were uncertain (Fig 1B). Again, a significantly lower proportion (*P*<0.001) of RUN (2.7%) perceived frequent running as detrimental for knee joint health compared with NRUN (23.1%) and NRUN-OA (24.2%). In HCP, 9.1% viewed running frequently as a risk factor for KOA (3.9% in running HCP; 17.6% in non-running HCP, *P*<0.001; Fig 2B).

From members of the public, 15.5% agreed that running marathons or longer distances would lead to KOA, and 43.6% were uncertain (Fig 1C). However, disagreement with that statement was significantly higher (*P*<0.001) in RUN (47.9%) than in NRUN (15.4%) and NRUN-OA (19.4%). Long-distance running was perceived as a risk factor for KOA by 22.2% of HCP (17.2% of running HCP; 30.4% of non-running HCP, *P* = 0.001; Fig 2C).

### Perception about running and progression of KOA

**Fig 3:**
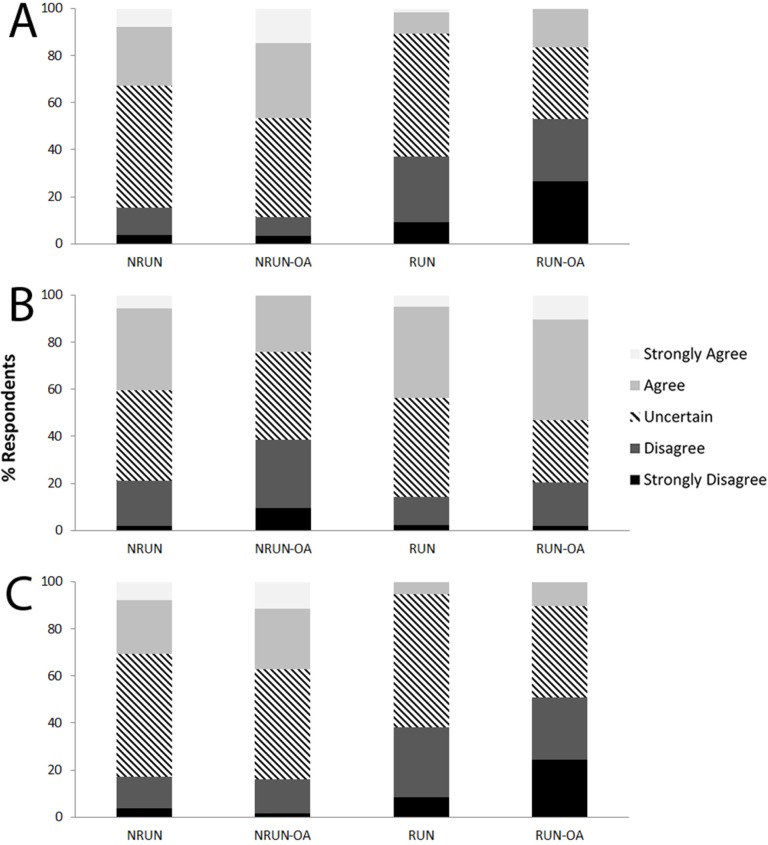
Perception of non-runners and runners about running and the progression of knee osteoarthritis. (A) “People with knee osteoarthritis who continue to run will sustain greater knee cartilage damage leading to more severe OA”; (B) “It is fine for people who have OA to run as long as they don’t have symptoms on the day they go running”; (C) “A person with knee osteoarthritis who keeps running regularly will speed up the need for joint replacement surgery (knee arthroplasty)”.

**Fig 4:**
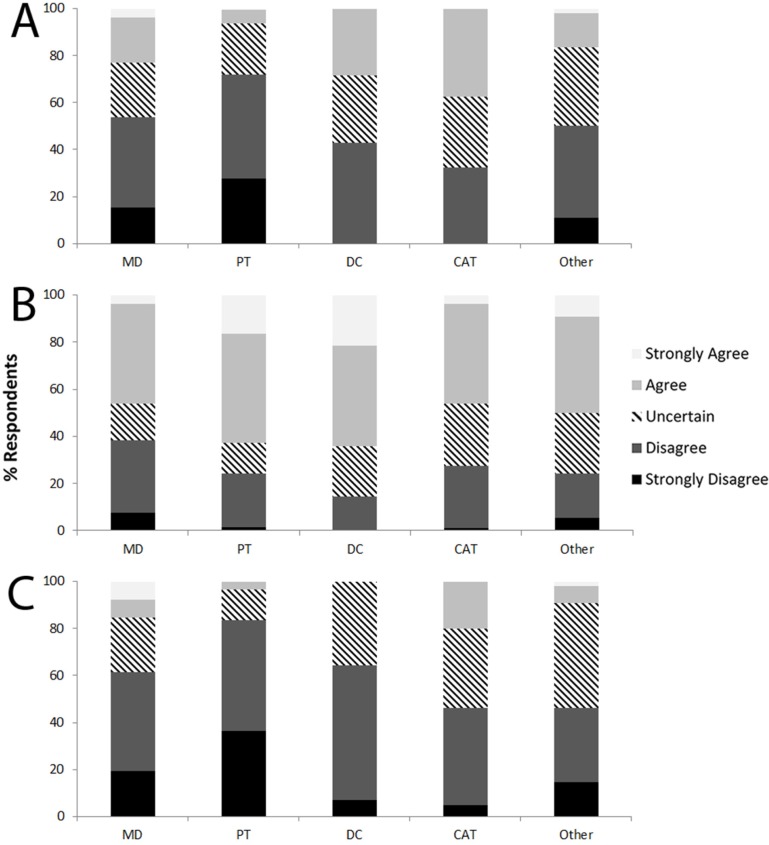
Perception of HCP about running and the progression of knee osteoarthritis. (A) “People with knee osteoarthritis who continue to run will sustain greater knee cartilage damage leading to more severe OA”; (B) “It is fine for people who have OA to run as long as they don’t have symptoms on the day they go running”; (C) “A person with knee osteoarthritis who keeps running regularly will speed up the need for joint replacement surgery (knee arthroplasty)”.

Overall, 17.9% of the members of the public answered that running with KOA would lead to greater knee joint damage, while 48.4% were uncertain (Fig 3A). A significantly greater proportion (*P*<0.004) of NRUN (32.7%) and NRUN-OA (46.8%) agreed with this statement than the RUN subgroup (10.7%). In HCP, 18.2% believed that running with KOA would lead to greater damage (9.6% of running HCP; 32.0% of non-running HCP, *P*<0.001), and overall 26.3% were uncertain (Fig 4A).

Running with KOA on days where there are no symptoms was seen as appropriate by 41.9% of members of the public, and 39.5% were uncertain (Fig 3B). A significantly greater proportion (*P*<0.001) of NRUN-OA respondents (38.7%) compared to RUN respondents (14.3%) thought it was not appropriate to do so. As for HCP, 55.2% agreed that running with KOA was appropriate when asymptomatic (54.8% of running HCP; 55.7% of non-running HCP, *P* = 0.270), and 18.5% were uncertain (Fig 4).

Finally, 12.4% of the general population perceived running with KOA as a means of accelerating the need for a total knee arthroplasty, while more than half were uncertain (53.1%; Fig 3C). NRUN (30.8%) and NRUN-OA (37.1%) agreed with that statement in a significantly greater proportion than RUN (5.4%). Only 9.4% of HCP agreed (4.1% of running HCP; 18.0% of non-running HCP, *P*<0.001), while 65.2% disagreed and 25.4% were uncertain (Fig 4C).

### Clinical recommendations of HCP

37.1% of HCP typically recommend their running patients with KOA to modify their running habits (34.3% of running HCP; 27.2% of non-running HCP, *P* = 0.663), while only 2.7% have recommended a majority (>50%) of running patients with KOA to quit running (1.0% of running HCP; 5.6% of non-running HCP, *P* = 0.107). Overall, 50.6% HCP reported that they now advise more runners with KOA to keep running than what they were previously recommending (57.3% of running HCP; 39.8% of non-running HCP, *P* = 0.007); 43.5% said they had not changed their recommendations over the years. Finally, 30.3% of HCP would recommend runners who sustained a total knee replacement to continue running post-operatively (31.2% of running HCP; 28.8% of non-running HCP, *P* = 0.713), but 39.4% were uncertain.

## Discussion

This study is the first to provide data on how the general public and HCP perceive running with respect to knee joint health, as well as their perceptions of the appropriateness of running in individuals with pre-existing KOA. Even though regular physical activity is strongly advocated for the prevention and management of KOA, beliefs specifically about running do not reflect such certainty and are in line with the current state of evidence. While people who run (both in the general public and HCP) were more positive to the effects of running on knee joint health, there was no clear direction overall to definitively state whether people in our survey perceived running as safe for knee joint health or not. Indeed, one of the most interesting findings is the relatively high proportion of respondents who were uncertain about running being appropriate or not for knee joint health.

In the general public, non-runners tended to perceive running as more detrimental than runners. While this may not be surprising, long distance running was still considered a risk factor for developing KOA by over 40% of respondents from the general public. Despite previous studies suggesting that running–even high mileage–may not be linked with KOA [18–21], a recent systematic review reported that a history of *recreational* running was associated with a lower prevalence of KOA (3.5%) when compared with controls (10.2%)[15]. Conversely, the authors found that a history of *competitive* running was associated with a higher prevalence of KOA (13.3%). When combined with our results, this outlines the need for future prospective studies to report detailed parameters about running habits, other than simply the level of competition. Indeed, clearer recommendations can be made to the public and HCP if more information is provided about the development of OA depending on running frequency, volume and speed.

Similarly, more than half of respondents from the general public were uncertain if running with pre-existing KOA would accelerate the need for a total knee arthroplasty. Previous studies have reported a potential protective effect of running against joint replacement surgery in those without knee [20] or hip [4] osteoarthritis. Even though a recent systematic review outlined that low to moderate impact therapeutic exercise programs were not detrimental to cartilage in those with KOA,[22] no studies investigated the odds of a surgical endpoint when running with pre-existing KOA. Only Lo et al. have suggested that running may not accelerate the progression of KOA in those with a diagnosis of KOA. However, it cannot be excluded that specific training parameters may be more detrimental to knee joint health than others. Indeed, the current body of literature fails to provide clear guidance about optimal frequency, distance or speed in runners with KOA, or whether any of these variables should be altered upon diagnosis. Thus, the high proportion of uncertainty is not totally surprising and clearly reflects a need for more research to orientate the population. Fear of causing pain or further degeneration could represent an important psychological barrier to physical activity uptake in individuals with KOA [9], which may lead to a more sedentary lifestyle. A number of factors may influence how one perceives the appropriateness of running with KOA, for example the level of symptoms, physical fitness, comorbidities, the importance given to running and experience from peers. In the current study, respondents from the RUN group predominantly reported (80.2%) that they would significantly decrease their level of running if they were to receive a diagnosis of KOA in the future. Responses from the RUN-OA group confirmed that a certain proportion of runners quit running or reduce training upon their diagnosis of KOA, hence potentially reducing beneficial effects of an active lifestyle [23]. Thus, our results outline the need for more research to guide both runners and non-runners with KOA in their quest for meaningful physical activity.

It has previously been suggested that only a small to moderate proportion of people with KOA meet physical activity guidelines [7]. Furthermore, even though a majority of individuals with KOA consult HCP for their symptoms, no more than half are prescribed exercises during these consultations [24, 25]. Since physical activity is a cornerstone of treatment for KOA [5, 6], and given that two thirds of respondents to our survey sought advice about running and knee joint health, better consensus on optimal clinical guidelines regarding running is needed to guide HCP. Conflicting recommendations from HCP can cause confusion in patients, which has been documented as a barrier to physical activity [17]. Despite most HCP not reporting negative beliefs about running and knee joint health in the current sample, a relatively high level of uncertainty was detected. This could potentially be attributed to a difficulty to generalize to all patients, given that clinical management requires an individualized approach. Such uncertainty can obviously affect recommendations and thus, activities chosen by the general public. There seemed to be a trend in HCP–potentially based on recent evidence that does not support the association between running and KOA [15, 19, 20, 26]–to recommend an increased number of runners with KOA to continue to enjoy running, albeit with some modifications based on symptoms. In the absence of clear guidelines in the literature, however, personal preferences and biases of HCP could also influence their perceptions and recommendations, as shown by the disparity between running and non-running HCP.

This study is not without limitations. First, fewer non-runners participated despite our original intent to recruit equally across all subgroups. An online survey about running and knee joint health may be more likely to be filled out by runners and HCP than by non-runners, and we believe that our statistical comparisons provide useful insight on the different perceptions between runners and non-runners in Canada. However, since it was impossible for us to keep track of all the people who have been in contact with study advertisements, we cannot assume that our findings are fully representative of the entire Canadian population. Second, no sex-specific analyses were conducted since the sample size in some subgroups were too small. Given the greater prevalence of KOA in females,[1] and potential discrepancies in perceptions between males and females, future studies should consider conducting separate analyses for both sexes. Third, data collected in this study is descriptive in nature, and using an online design may be subject to response bias. For example, it is impossible to ascertain that respondents really received a diagnosis of KOA, or that they were indeed HCP. However, this is an intrinsic limitation of any self-reported online survey. Fourth, it remains unclear is respondents who reported decreasing or quitting running after getting a diagnosis of KOA replaced running by other activities. Thus, we can`t ascertain whether these individuals maintained benefits of physical activity by means other than running. Finally, we conducted the survey among residents of Canada only. This choice was made to provide a clearer representation of local beliefs, and considering the over-representation of healthy runners in our sample, results may not be generalizable to all populations. Future studies investigating similar constructs in other countries may need to translate and cross-culturally adapt questions to the targeted population.

## Conclusions

In conclusion, the general public and HCP in Canada reported high rates of uncertainty regarding running as a risk factor to develop KOA, and about the appropriateness of running with pre-existing KOA. Considering efforts to provide recommendations of meaningful regular physical activity to promote knee joint health, results from this study emphasize the need for additional research specifically investigating running, as well as the development of future knowledge translation strategies to the general public and HCP. High-quality prospective studies are warranted both in cohorts of individuals with and without KOA. Importantly, caution must be taken to monitor detailed training parameters such as frequency, speed and distance, so that clearer recommendations can be issued on optimal dosage for knee joint health and tailored to individual patients with KOA.

## Supporting information

S1 File Survey questions for respondents from each subgroup, and associated response choices. Subgroups are denoted by numbers as follows: 1 = NRUN; 2 = NRUN-OA; 3 = RUN; 4 = RUN-OA; 5 = HCP.
(DOCX)

S2 File Full de-identified dataset. (DOCX)

S3 File Detailed results for subgroups of non-runners and runners. (DOCX)
