# Supplementary material for: What are the perceptions about running and knee joint health among the public and healthcare practitioners in Canada?
Source: PLoS One. 2018 Oct 1;13(10):e0204872. doi: 10.1371/journal.pone.0204872 (PMC6166953; doi:10.1371/journal.pone.0204872)
Supplement: S1 File — Subgroups are denoted by numbers as follows: 1 = NRUN; 2 = NRUN-OA; 3 = RUN; 4 = RUN-OA; 5 = HCP. (DOCX) [file pone.0204872.s001.docx]

**S1 File. Survey questions for respondents from each subgroup, and associated response choices.** Subgroups are denoted by numbers as follows: 1=NRUN; 2=NRUN-OA; 3=RUN; 4=RUN-OA; 5=HCP.

| **SUBGROUPS** | **QUESTION** | **CHOICES** |
| --- | --- | --- |
| 1,2,3,4,5 | What is your age? | N/A |
| 1,2,3,4,5 | What is your sex? | - Female - Male |
| 1,2,3,4,5 | Please enter the first 3 values of your postal code in Canada. | N/A |
| 1,2,3,4,5 | What is the highest level of education you have completed? | - Less than high school completion - High School - Trades certificate, vocational school diploma, apprenticeship - Non-university certificate below Bachelor's level - Bachelor’s degree - Masters degree - Doctorate degree |
| 1,2,3,4,5 | What is your current height and weight? | N/A |
| 1,2,3,4,5 | In general, would you say your health is | - Excellent - Very good - Good - Fair - Poor |
| 1,2,3,4,5 | Compared to one year ago, how would you rate your overall health now? | - Much better now - Somewhat better now - About the same - Somewhat worse now - Much worse now |
| 1,2,3,4,5 | Have you ever had a torn knee ligament or major knee injury? Check all that apply. | - No, I have never sustained a significant traumatic injury to my knee - Yes, my anterior cruciate ligament (ACL) - Yes, my posterior cruciate ligament (PCL) - Yes, my medial collateral ligament (MCL) - Yes, my lateral collateral ligament (LCL) - Yes, my meniscus (medial or lateral) - Yes, fractured my knee - Yes, but I am not sure what structure was injured |
| 1,2,3,4,5 | Compared to one year ago, how would you rate your current level of physical activity? | - Much more active now - Somewhat more active now - About the same level of activity - Somewhat less active now - Much less active now |
| 1,2,3,4,5 | In which recreational activities do you currently regularly participate (once or more per week)? Check all that apply. | - Walking - Cycling - Swimming - Team sports (hockey, soccer, basketball, volleyball, etc.) - Yoga, Tai Chi - Other, please specify - I do not participate in any recreational activities |
| 1,2,3,4,5 | In general, I see regular running as an activity that hurts the knee joint. | - I strongly disagree - I disagree - Uncertain - I agree - I strongly agree |
| 1,2,3,4,5 | Frequent running can lead to getting knee osteoarthritis. | - I strongly disagree - I disagree - Uncertain - I agree - I strongly agree |
| 1,2,3,4,5 | Running long distances (such as marathons and ultra-marathons) can lead to getting knee osteoarthritis. | - I strongly disagree - I disagree - Uncertain - I agree - I strongly agree |
| 1,2,3,4,5 | People with knee osteoarthritis who continue to run will sustain greater knee cartilage damage leading to more severe osteoarthritis. | - I strongly disagree - I disagree - Uncertain - I agree - I strongly agree |
| 1,2,3,4,5 | It is fine for people who have osteoarthritis to run as long as they don’t have symptoms on the day they go running. | - I strongly disagree - I disagree - Uncertain - I agree - I strongly agree |
| 1,2,3,4,5 | A person with knee osteoarthritis who keeps running regularly will speed up the need for joint replacement surgery. | - I strongly disagree - I disagree - Uncertain - I agree - I strongly agree |
| 1,2,3,4 | Please let us know what advice you have received about running for joint health. | - N/A |
| 1,2,3,4 | Who provided you with the advice on running and joint health? Check all that apply. | - Family physician - Rheumatologist - Sports medicine physician - Orthopaedic surgeon - Physiotherapist - Chiropractor - Athletic therapist - Friend, colleague or family member - Public forums or presentations - Internet - Television - Radio - Print media (newspapers/magazines/books) - Scientific literature - Other, please specify - I haven’t received any advice on running and knee joint health |
| 1,2 | You said that you aren’t regularly running right now. Have you been a regular runner in the past? | - No - Yes, but I stopped because of injuries to my knees - Yes, but I stopped because of my diagnosis of knee osteoarthritis *(choice only available to subgroup 2)* - Yes, but I stopped because of injuries other than my knees - Yes, but I stopped because of other reasons |
| 1 | One of the reasons I don’t run regularly is to avoid getting osteoarthritis in my knees. | - I strongly disagree - I disagree - Uncertain - I agree - I strongly agree |
| 2 | Do you use or have used medications to help your knee feel better? | - Yes, non-steroidal anti-inflammatory medications (for instance: Ibuprofen or Advil, Naproxen or Naprosyn, Diclofenac or Voltaren, Celebrex, etc.) - Yes, steroidal anti-inflammatory medications (for instance: cortisone, prednisone, etc.) - Yes, viscosupplementation (Hyaluronic acid such as Synvisc, Monovisc, Neovisc, etc.) - I don’t use or haven’t used medications to help my knee feel better |
| 4 | Do you use or have used medications to help your knee feel better when running? | - Yes, non-steroidal anti-inflammatory medications (for instance: Ibuprofen or Advil, Naproxen or Naprosyn, Diclofenac or Voltaren, Celebrex, etc.) - Yes, steroidal anti-inflammatory medications (for instance: cortisone, prednisone, etc.) - Yes, viscosupplementation (Hyaluronic acid such as Synvisc, Monovisc, Neovisc, etc.) - I don’t use or haven’t used medications to help my knee feel better when running |
| 3,4 | How many years have you been running regularly (cumulative in your life)? | - Less than 1 year - At least 1 year, but less than 3 years - At least 3 years, but less than 5 years - At least 5 years, but less than 10 years - At least 10 years, but less than 20 years - 20 years and more |
| 3,4 | On average, how many kilometers do you run per week? (average last 6 months) | - Less than 10 kilometers - Between 10 and 19 kilometers - Between 20 and 29 kilometers - Between 30 and 49 kilometers - Between 50 and 100 kilometers - More than 100 kilometers |
| 3,4 | What is the longest distance you have ever run in one day/session? | - 5 km - 10 km - Half-marathon (21.1 km) - Marathon (42.2 km) - Ultra-marathon (over 50 km) |
| 3,4 | On average, how many times do you run per week? (average last 6 months) | - 1 - 2 - 3 - 4 - 5 - 6 - 7 - More than 7 |
| 3,4 | Are you part of a running club or group? | - Yes - No |
| 3,4 | How often do you run speed intervals? (training sessions involving bursts of running at significantly higher speed than your comfortable running speed) | - Never - Less than once per week - Once per week - Twice per week - More than twice per week |
| 3 | If I were to develop knee pain (but without a diagnosis of knee osteoarthritis), I would… (check all that apply) | - Stop running - Run less frequently - Run less distance - Run slower - Not change my running habits |
| 3 | If I was diagnosed with knee osteoarthritis by a doctor, I would… (check all that apply) | - Stop running - Run less frequently - Run less distance - Run slower - Not change my running habits |
| 4 | When I developed knee pain (but without knowing that I had osteoarthritis), I… (check all that apply) | - Stopped running temporarily - Decreased my running frequency (number of times per week) - Decreased my running distance - Decreased my running speed - Did not change my running habits |
| 4 | When I was diagnosed with knee osteoarthritis, I… (check all that apply) | - Stopped running temporarily - Decreased my running frequency (number of times per week) - Decreased my running distance - Decreased my running speed - Did not change my running habits |
| 5 | Have you been diagnosed with knee osteoarthritis by a healthcare professional? | - Yes - No |
| 5 | How would you best describe your running habits? | - I currently run regularly (once or more per week) - I currently run, but infrequently (less than 4 times per month) - I don’t currently run regularly (once or more per week), but I have in the past - I have never run regularly or infrequently |
| 5 | What is your profession? | - Medical doctor (MD) - Physiotherapist (PT) - Chiropractor (DC) - Athletic therapist (CAT) - Other, please specify |
| 5 | Do you have an active license to practice? | - Yes, and I work clinically full-time - Yes, and I work clinically part-time - Yes, but I don’t currently work as a clinician (research, administrative position) - No (including maternity leave) - I am retired |
| 5 | What is your primary area of practice? | - Orthopaedics - Rheumatology - General practice - Sports medicine - Other, please specify |
| 5 | How many years have you been practicing as a healthcare professional? | - Less than 1 year - At least 1 year, but less than 3 years - At least 3 years, but less than 5 years - At least 5 years, but less than 10 years - At least 10 years, but less than 20 years - 20 years and more |
| 5 | What percentage of your patients who are runners with knee osteoarthritis have you recommended to modify their running habits? | - 0% - 1-25% - 26-50% - 51-75% - 76-99% - 100% |
| 5 | What percentage of your patients who are runners with knee osteoarthritis have you recommended to quit running? | - 0% - 1-25% - 26-50% - 51-75% - 76-99% - 100% |
| 5 | Have your recommendations on running and knee osteoarthritis changed over the course of your career? | - No - Yes, I now advise more runners with knee OA to keep running - Yes, I now advise fewer runners with knee OA to keep running |
| 5 | If a patient presents to you following a total knee joint replacement surgery and wants to continue running, how likely are you to recommend that person to continue running? | - Not at all - Unlikely - Uncertain - Likely - Definitely |
