# Supplementary material for: What are the perceptions about running and knee joint health among the public and healthcare practitioners in Canada?
Source: PLoS One. 2018 Oct 1;13(10):e0204872. doi: 10.1371/journal.pone.0204872 (PMC6166953; doi:10.1371/journal.pone.0204872)
Supplement: S2 File — (DOCX) [file pone.0204872.s002.docx]

**S2 File. Detailed results for subgroups of runners and non-runners.**

**In general, I see regular running as an activity that hurts the knee joint.**

|  | **NRUN** | | **NRUN, OA** | | **RUN** | | **RUN, OA** | |
| --- | --- | --- | --- | --- | --- | --- | --- | --- |
|  | n | % [95% C.I.] | n | % [95% C.I.] | n | % [95% C.I.] | n | % [95% C.I.] |
| **Strongly Disagree** | 3 | 5.8 [2.0, 15.6] | 4 | 6.5 [2.5, 15.5] | 103 | 30.5 [25.8, 35.6] | 13 | 26.0 [15.9, 39.6] |
| **Disagree** | 13 | 25.0 [15.2, 38.2] | 12 | 19.4 [11.4, 30.9] | 143 | 42.3 [37.2, 47.6] | 15 | 30.0 [19.1, 43.8] |
| **Uncertain** | 14 | 26.9 [16.8, 40.3] | 22 | 35.5 [24.7, 47.9] | 78 | 23.1 [18.9, 27.9] | 16 | 32.0 [20.8, 45.8] |
| **Agree** | 17 | 32.7 [21.5, 46.2] | 18 | 29.0 [19.2, 41.3] | 14 | 4.1 [2.5, 6.8] | 6 | 12.0 [5.6, 23.8] |
| **Strongly Agree** | 5 | 9.6 [4.2, 20.6] | 6 | 9.7 [4.5, 19.6] | 0 | 0.0 [0.0, 1.1] | 0 | 0.0 [0.0, 7.1] |

**Frequent running can lead to getting knee osteoarthritis.**

|  | **NRUN** | | **NRUN, OA** | | **RUN** | | **RUN, OA** | |
| --- | --- | --- | --- | --- | --- | --- | --- | --- |
|  | n | % [95% C.I.] | n | % [95% C.I.] | n | % [95% C.I.] | n | % [95% C.I.] |
| **Strongly Disagree** | 3 | 5.8 [2.0, 15.6] | 2 | 3.2 [0.9, 11.0] | 96 | 28.4 [23.9, 33.4] | 13 | 26.0 [15.9, 39.6] |
| **Disagree** | 11 | 21.2 [12.2, 34.0] | 16 | 25.8 [16.6, 37.9] | 135 | 39.9 [34.9, 45.3] | 18 | 36.0 [24.1, 49.9] |
| **Uncertain** | 26 | 50.0 [36.9, 63.1] | 29 | 46.8 [34.9, 59.0] | 98 | 29.0 [24.4, 34.0] | 17 | 34.0 [22.4, 47.9] |
| **Agree** | 7 | 13.5 [6.7, 25.3] | 11 | 17.7 [10.2, 29.0] | 9 | 2.7 [1.4, 5.0] | 2 | 4.0 [3.2, 18.8] |
| **Strongly Agree** | 5 | 9.6 [4.2, 20.6] | 4 | 6.5 [2.5, 15.5] | 0 | 0.0 [0.0, 1.1] | 0 | 0.0 [0.0, 7.1] |

**Running long distances (such as marathons and ultra, marathons) can lead to getting knee osteoarthritis.**

|  | **NRUN** | | **NRUN, OA** | | **RUN** | | **RUN, OA** | |
| --- | --- | --- | --- | --- | --- | --- | --- | --- |
|  | n | % [95% C.I.] | n | % [95% C.I.] | n | % [95% C.I.] | n | % [95% C.I.] |
| **Strongly Disagree** | 3 | 5.8 [2.0, 15.6] | 2 | 3.2 [0.9, 11.0] | 60 | 17.8 [14.1, 22.2] | 5 | 10.0 [4.4, 21.4] |
| **Disagree** | 5 | 9.6 [4.2, 20.6] | 10 | 16.1 [9.0, 27.2] | 102 | 30.2 [25.5, 35.3] | 18 | 36.0 [24.1, 49.9] |
| **Uncertain** | 30 | 57.7 [44.2, 70.1] | 28 | 45.2 [33.4, 57.5] | 144 | 42.6 [37.4, 47.9] | 17 | 34.0 [22.4, 47.9] |
| **Agree** | 9 | 17.3 [9.4, 29.7] | 14 | 22.6 [14.0, 34.4] | 29 | 8.6 [6.0, 12.1] | 10 | 20.0 [11.2, 33.0] |
| **Strongly Agree** | 5 | 9.6 [4.2, 20.6] | 8 | 12.9 [6.7, 23.5] | 3 | 0.9 [0.3, 2.6] | 0 | 0.0 [0.0, 7.1] |

**People with knee osteoarthritis who continue to run will sustain greater knee cartilage damage leading to more severe osteoarthritis.**

|  | **NRUN** | | **NRUN, OA** | | **RUN** | | **RUN, OA** | |
| --- | --- | --- | --- | --- | --- | --- | --- | --- |
|  | n | % [95% C.I.] | n | % [95% C.I.] | n | % [95% C.I.] | n | % [95% C.I.] |
| **Strongly Disagree** | 2 | 3.9 [1.1, 13.0] | 2 | 3.2 [0.9, 11.0] | 31 | 9.2 [6.6, 12.8] | 13 | 26.5 [16.2, 40.3] |
| **Disagree** | 6 | 11.5 [5.4, 23.0] | 5 | 8.1 [3.5, 17.5] | 94 | 27.8 [23.5, 33.0] | 13 | 26.5 [16.2, 40.3] |
| **Uncertain** | 27 | 51.9 [38.7, 64.9] | 26 | 41.9 [30.5, 54.3] | 175 | 52.1 [46.8, 57.4] | 15 | 30.6 [19.5, 44.5] |
| **Agree** | 13 | 25.0 [15.2, 38.2] | 20 | 32.3 [22.0, 44.6] | 31 | 9.2 [6.6, 12.8] | 8 | 16.3 [8.5, 29.0] |
| **Strongly Agree** | 4 | 7.7 [3.0, 18.2] | 9 | 14.5 [7.8, 25.4] | 5 | 1.5 [0.6, 3.4] | 0 | 0.0 [0.0, 7.3] |

**It is fine for people who have osteoarthritis to run as long as they don’t have symptoms on the day they go running.**

|  | **NRUN** | | **NRUN, OA** | | **RUN** | | **RUN, OA** | |
| --- | --- | --- | --- | --- | --- | --- | --- | --- |
|  | n | % [95% C.I.] | n | % [95% C.I.] | n | % [95% C.I.] | n | % [95% C.I.] |
| **Strongly Disagree** | 1 | 1.9 [0.3, 10.1] | 6 | 9.7 [4.5, 19.6] | 8 | 2.4 [1.2, 4.6] | 1 | 2.0 [3.6, 10.7] |
| **Disagree** | 10 | 19.2 [10.8, 31.9] | 18 | 29.0 [19.2, 41.3] | 40 | 11.8 [8.9, 15.8] | 9 | 18.4 [10.0, 31.4] |
| **Uncertain** | 20 | 38.5 [26.5, 52.0] | 23 | 37.1 [26.2, 49.6] | 141 | 42.0 [36.8, 47.3] | 13 | 26.5 [16.2, 40.3] |
| **Agree** | 18 | 34.6 [23.2, 48.2] | 15 | 24.2 [15.2, 36.2] | 131 | 39.0 [33.9, 44.3] | 21 | 42.9 [30.0, 56.7] |
| **Strongly Agree** | 3 | 5.8 [2.0, 15.6] | 0 | 0.0 [0.0, 5.8] | 16 | 4.8 [3.0, 7.6] | 5 | 10.2 [4.4, 21.8] |

**A person with knee osteoarthritis who keeps running regularly will speed up the need for joint replacement surgery.**

|  | **NRUN** | | **NRUN, OA** | | **RUN** | | **RUN, OA** | |
| --- | --- | --- | --- | --- | --- | --- | --- | --- |
|  | n | % [95% C.I.] | n | % [95% C.I.] | n | % [95% C.I.] | n | % [95% C.I.] |
| **Strongly Disagree** | 2 | 3.9 [1.1, 13.0] | 1 | 1.6 [0.3, 8.6] | 28 | 8.3 [5.8, 11.8] | 12 | 24.5 [14.6, 38.1] |
| **Disagree** | 7 | 13.5 [6.7, 25.3] | 9 | 14.5 [7.8, 25.4] | 100 | 29.8 [25.1, 34.9] | 13 | 26.5 [16.2, 40.3] |
| **Uncertain** | 27 | 51.9 [38.7, 64.9] | 29 | 46.8 [34.9, 59.0] | 190 | 56.6 [51.2, 61.8] | 19 | 38.8 [26.4, 52.8] |
| **Agree** | 12 | 23.1 [13.7, 36.1] | 16 | 25.8 [16.6, 37.9] | 17 | 5.1 [3.2, 8.0] | 5 | 10.2 [4.4, 21.8] |
| **Strongly Agree** | 4 | 7.7 [3.0, 18.2] | 7 | 11.3 [5.6, 21.5] | 1 | 0.3 [0.1, 1.9] | 0 | 0.0 [0.0, 7.3] |
