# Supplementary material for: What are the perceptions about running and knee joint health among the public and healthcare practitioners in Canada?
Source: PLoS One. 2018 Oct 1;13(10):e0204872. doi: 10.1371/journal.pone.0204872 (PMC6166953; doi:10.1371/journal.pone.0204872)
Supplement: S3 File — (DOCX) [file pone.0204872.s003.docx]

**S3 File. Detailed results for HCP**

**In general, I see regular running as an activity that hurts the knee joint.**

|  | **Running HCP** | | **Non-running HCP** | |
| --- | --- | --- | --- | --- |
|  | n | % [95% C.I.] | n | % [95% C.I.] |
| **Strongly Disagree** | 94 | 46.1 [39.4, 52.9] | 35 | 28.7 [21.4, 37.3] |
| **Disagree** | 83 | 40.7 [34.2, 47.5] | 44 | 36.1 [28.1, 44.9] |
| **Uncertain** | 19 | 9.3 [6.0, 14.1] | 25 | 20.5 [14.3, 28.5] |
| **Agree** | 7 | 3.4 [1.7, 6.9] | 18 | 14.8 [9.5, 22.1] |
| **Strongly Agree** | 1 | 0.5 [0.1, 2.7] | 0 | 0.0 [0.0, 3.1] |

**Frequent running can lead to getting knee osteoarthritis.**

|  | **Running HCP** | | **Non-running HCP** | |
| --- | --- | --- | --- | --- |
|  | n | % [95% C.I.] | n | % [95% C.I.] |
| **Strongly Disagree** | 86 | 42.2 [35.6, 49.0] | 24 | 19.7 [13.6, 27.6] |
| **Disagree** | 94 | 46.1 [39.4, 52.9] | 48 | 39.3 [31.1, 48.2] |
| **Uncertain** | 16 | 7.8 [4.9, 12.4] | 30 | 24.6 [17.8, 32.9] |
| **Agree** | 8 | 3.9 [2.0, 7.5] | 20 | 16.4 [10.9, 24.0] |
| **Strongly Agree** | 0 | 0.0 [0.0, 1.9] | 0 | 0 [0.0, 3.1] |

**Running long distances (such as marathons and ultra, marathons) can lead to getting knee osteoarthritis.**

|  | **Running HCP** | | **Non-running HCP** | |
| --- | --- | --- | --- | --- |
|  | n | % [95% C.I.] | n | % [95% C.I.] |
| **Strongly Disagree** | 43 | 21.1 [16.0, 27.2] | 11 | 9.0 [5.1, 15.4] |
| **Disagree** | 76 | 37.3 [30.9, 44.1] | 36 | 29.5 [22.1, 38.1] |
| **Uncertain** | 50 | 24.5 [19.1, 30.9] | 38 | 31.2 [23.6, 39.8] |
| **Agree** | 32 | 15.7 [11.3, 21.3] | 35 | 28.7 [21.4, 37.3] |
| **Strongly Agree** | 3 | 1.5 [0.5, 4.2] | 2 | 1.6 [0.5, 5.8] |

**People with knee osteoarthritis who continue to run will sustain greater knee cartilage damage leading to more severe osteoarthritis.**

|  | **Running HCP** | | **Non-running HCP** | |
| --- | --- | --- | --- | --- |
|  | n | % [95% C.I.] | n | % [95% C.I.] |
| **Strongly Disagree** | 40 | 20.3 [15.3, 26.5] | 10 | 8.4 [4.6, 14.8] |
| **Disagree** | 80 | 40.6 [34.0, 47.6] | 46 | 38.7 [30.4, 47.6] |
| **Uncertain** | 58 | 29.4 [23.5, 36.2] | 26 | 21.9 [15.4, 30.1] |
| **Agree** | 19 | 9.6 [6.3, 14.6] | 34 | 28.6 [21.2, 37.3] |
| **Strongly Agree** | 0 | 0.0 [0.0, 1.9] | 3 | 2.5 [0.9, 7.2] |

**It is fine for people who have osteoarthritis to run as long as they don’t have symptoms on the day they go running.**

|  | **Running HCP** | | **Non-running HCP** | |
| --- | --- | --- | --- | --- |
|  | n | % [95% C.I.] | n | % [95% C.I.] |
| **Strongly Disagree** | 5 | 2.5 [1.1, 5.8] | 3 | 2.5 [0.9, 7.2] |
| **Disagree** | 51 | 25.9 [20.3, 32.4] | 23 | 19.3 [13.2, 27.3] |
| **Uncertain** | 33 | 16.8 [12.2, 22.6] | 27 | 22.7 [16.1, 31.0] |
| **Agree** | 79 | 40.1 [33.5, 47.1] | 59 | 49.6 [40.8, 58.4] |
| **Strongly Agree** | 29 | 14.7 [10.5, 20.3] | 7 | 5.9 [2.9, 11.6] |

**A person with knee osteoarthritis who keeps running regularly will speed up the need for joint replacement surgery.**

|  | **Running HCP** | | **Non-running HCP** | |
| --- | --- | --- | --- | --- |
|  | n | % [95% C.I.] | n | % [95% C.I.] |
| **Strongly Disagree** | 55 | 27.9 [22.1, 34.6] | 16 | 13.5 [8.5, 20.7] |
| **Disagree** | 89 | 45.2 [38.4, 52.2] | 47 | 39.5 [31.2, 48.5] |
| **Uncertain** | 45 | 22.8 [17.5, 29.2] | 36 | 30.3 [22.7, 39.0] |
| **Agree** | 8 | 4.1 [2.1, 7.8] | 17 | 14.3 [9.1, 21.7] |
| **Strongly Agree** | 0 | 0.0 [0.0, 1.9] | 3 | 2.5 [0.9, 7.2] |
